# Progressive disability in elderly population among tribals of Telangana: a cross sectional study

**DOI:** 10.1186/s12939-017-0600-4

**Published:** 2017-06-19

**Authors:** Ajitha Katta, Anil Kumar Indira Krishna, Bagavandas M, Tomofumi Anegawa, Suresh Munuswamy

**Affiliations:** 1 SRM School of Public Health, SRM University, Kattankulathur, Chennai, India; 2 Keio Business School, Keio University, Hiyoshi, Minato, Japan; 3 DST Health Informatics Rapid Design Lab, Hyderabad, India; 4 PHFI-Indian Institute of Public Health, Hyderabad, India

**Keywords:** Disability, Progressive, Elderly, Tribal, Telangana, India

## Abstract

**Background:**

The tribal population of Telangana, India, lives in remote and difficult conditions. This study was carried out to find out estimate, the prevalence and progression of disability in elderly population among tribals of Khammam District, Telangana state, India.

**Methods:**

A population based cross sectional survey was conducted in villages of Tribal Sub Plan area. Elderly people who are 60 years or older were chosen with a two stage sampling procedure: (1) probability proportion to size was used to select clusters and (2) in each selected cluster households were selected by systematic random sampling. The participants were interviewed with the 36 item Telugu version of the World Health Organization Disability Assessment Schedule (WHODAS 2.0) questionnaire. Socio- demographic information, behavioral measurements, health and social benefit indicators were also assessed. Descriptive analytical methods were used for prevalence estimation and logistic regression was used to examine the associations of progressive age over disability among elderly.

**Results:**

A total of 506 elderly people from 1349 households in 20 villages across 31mandals of Khammam were interviewed. Majority of elderly population among tribals were illiterate (men 88.94%; women 99.33%), used tobacco (men 81.25%; women 57.72%), consumed alcohol (men 80.77%; women 47.32%) and were hypertensive (men 53.85%; women 63.42%). The prevalence of disability was higher in women. Maximum disability in the interviewed elderly population was seen in domains of performing house hold activities, and mobility. In comparison with men, women expressed more disability for majority of domains. As age progressed, the disability for self-care domain increased to a maximum of 2.6 times in men and 6.6 times in women and for mobility domain increased to a maximum of 9.7 times in men and 7.2 times in women.

**Conclusions:**

Although present disability modifying mobility Assistive Devices (AD) can help elderly in overcoming disability, these are primarily designed for built environments. As the needs, cultural sensitivities, and living environment of elderly population in tribals are unique, newer innovative assistive devices should be designed and developed.

## Background

The world’s population is ageing rapidly. People aged 60 and above are defined as elderly and the population of global elderly is projected to increase from 12.3% at present to 22% by 2050 [1]. Disability in elderly is understood as the effect of progressive unwinding of human homeostatic equilibrium [2, 3]. As disability increases with age, it is imperative to understand the nature of its progress and design the health and social care service systems based on the needs of the elderly disabled population [4, 5]. Disability is defined clinically and functionally. The clinical definition of disability focuses on bodily impairment while the functional definition focuses on limitation in the Activities of Daily Living (ADL) at personal level and restriction in carrying out family or broader roles at social level [6]. Elderly can experience disability at functional level irrespective of the presence or absence of clinical disability. Screening studies with World Health Organization Disability.

Assessment Schedule-version 2.0 (WHODAS 2.0) questionnaire based assessment tool have shown that majority of elderly people have at least one or more ADL limitation.

Newer understanding and newer approaches may be necessary as elderly disabled people require significantly more health and social care resources to maintain acceptable quality of life. Health systems of several countries have already begun their refocus [7, 8].

The new state of Telangana was formed in 2014 by the reorganization of Andhra Pradesh, one of the largest states of India; in response to sustained agitation demanding improved, equitable development and better employment. Improving the lives of its 9.34% tribal population (as per 2011 census) [9] has also been proclaimed as a priority focus area. Article 355 (25) of the constitution of India describes scheduled tribes as “Communities of people with indications of primitive traits, distinctive culture, geographical isolation, shyness of contact with the community at large, and backwardness. The tribal populations in India face a multitude of issues including extreme levels of health and social care access deprivation [10]. Economic, cultural, social and developmental changes have been slow to affect, the geographically isolated tribal population in India. Hence in comparison with the rest of the country population, the tribal population has higher levels of illiteracy, and seemingly live in primitive conditions that are hostile, disease prone, lack sanitation, safe water and access to health care facilities. Regions that have high proportion of tribal populations like Telangana have been the focus of affirmative and supportive governmental action [10, 11]; however this approach has met with limited success. The tribal population primarily lives on subsistence collection of forest produce and shifting agriculture.

The ten existing districts of Telangana have further been re-organized into thirty one districts [12] and improved health care services are being rolled out [13]. As part of direct affirmative measures through conditional cash transfer schemes, the Government of Telangana provides regular monthly pensions of Indian Rupees (INR) 1000 (equivalent to United States Dollar 15) to all elderly population aged 65 and above and INR 1500 (USD 23) to all disabled people among tribals irrespective of age [14]. A new scheme to provide assistance to disabled persons for purchase of assistive devices has also been recently started by the government. The complimentary process of strengthening and refocusing of the health and social systems to support the needs of elderly people, people with disability, and their increasing burden of disease among tribals would require in-depth ground level understanding.

The first objective of this study was to estimate the prevalence of disability in terms of activity limitations and participation restrictions in elderly population among tribals. The second objective was to find out whether the disability was progressive with age and to quantify the progression. According to the 2011 Census, with in the state of Telangana, Khammam District had the highest percentage of Scheduled Tribes at 27% [9]. The district had 46 mandals or administrative divisions of a district, out of which 29 contiguous mandals and 2 mandals partly were covered under the Tribal Sub Plan (TSP) area [15]. TSP’s are one more aspect of joint federal and state government’s affirmative action plan to accelerate infrastructure development and income generation in predominantly tribal areas [16].

## Methods

A cross sectional population based survey was conducted among elderly population aged 60 years and above, in the TSP area of Khammam District, Telangana state. Two stage cluster sampling was used, in the first stage, all 904 villages of the TSP area were listed and 125 villages that have population of 500 and above and with 80% tribal population were selected. From among the 125 select villages, 20 clusters were randomly selected using probability proportion to size (PPS) sampling method. Each village was considered as a cluster for operational purposes. In the second stage, 72 households were selected from each selected cluster by using systematic random sampling method. Considering, 22% as prevalence of disability in elderly [17], a relative precision of 20%, non-response rate of 10% and design effect of 1.5, the final sample size of 580 elderly people was arrived at. The sampling frame of selected villages with information on individuals age details were not available; hence households were considered to select study subjects for the survey. At house hold level, the rural tribal family size was 5.4 [18] and the proportion of elderly people was 7.5% [19]. The number of households required to interview 580 elderly people was calculated at 1432. Of the estimated 1432 households, only 1349 households were surveyed under study. Remaining households were either locked or no adult respondent was available at household at the time of survey. Out of estimated 580, 506 elderly people were available at the time of data collection and participated in the study, giving a response rate at 87.2%. Nonresponse was largely due to their unavailability even after two repeated visits at two different dates and time. House to house, survey was conducted to select study participants. The inclusion criteria for eligible study participants were those who had resided in the Tribal Sub Plan (TSP) area for the previous 12 months and who were aged 60 years and above. Age was verified based on their identity card. Field study was conducted from December 2013 to June 2014.

As the objective of this study was to assess daily activity limitations and broader social participation restrictions of elderly individuals irrespective of clinical disability status, the WHO DAS 2.0 (Disability Assessment Schedule) 36-item version interviewer administered questionnaire was used. WHO DAS 2.0 Questionnaire was translated into local language i.e. Telugu, by a professional translator and the same was back translated into English by another independent translator by using WHO translation and back translation protocol [20]. The Telugu language questionnaire was pretested to test the clarity of the questions and ease of answering. Pilot study was conducted among 50 elderly people from randomly selected households in tribal areas for validating the questionnaire.

### Description of WHO DAS 2.0 36 - item version

WHO DAS 2.0 (36 item) [21] assesses disability from self-rated responses (based on level of difficulty in the last 30 days) to questions on level of functioning on 36 different items/areas of life. The questions are grouped in to six domains: (1) Cognition- understanding and communicating; (2) Mobility– moving & getting around; (3) Self-care-hygiene, dressing, eating & staying alone; (4) Getting along-interacting with other people; (5) Life activities-domestic responsibilities, leisure, work & school and (6) Participation-joining in community activities. Self-reported responses to each of the activity/items are graded on a 5-point Likert format (1: none; 5: extreme) by the interviewer based on the level of difficulty experienced by the study participants. For simplicity of analysis the responses were recoded to a dichotomous yes or no scale, with the responses “mild”, “moderate”, “severe” and “extreme/cannot do” all merged into a single positive response of “yes”. Disability was assessed by measuring the functioning based on the difficulty of carrying out different items across six domains. A proxy version of WHO DAS 2.0 questionnaire was administered to participants who were unable to respond to questions due to cognitive or motor disabilities. In case of participants who were unable to respond to questions due to cognitive or motor disabilities, WHO DAS 2.0 (36 item) proxy version questionnaire was administered to participants. Item D4.4 (making new friends), was excluded owing to an unusually high proportion of missing values. As the majority of the study participants were not gainfully employed through traditional means of employment and lived on subsistence collection of forest produce, the Items from D5.5 to D5.5, (work activities), were excluded.

### Socio demographic and health status Assessment

WHO STEPwise approach to Surveillance (STEPS) questionnaire was used to asses risk factors for general state of health. The interviewer administered assessment questions can be grouped in to three focus areas: (1) Socio demographic data including age, gender, marital status, education and living arrangements; (2) behavioral measurements like tobacco and alcohol use and; (3) assessment of health indicators like self-rated health and hypertension. In addition, information on social benefit indicators like visit by health worker and receipt of old age pension from government were collected from self-reported responses to interviewer administered survey modelled on WHO STEPS questionnaire [22]. Anthropometric measurements (height and weight) and blood pressure [BP] were recorded in all interviewed elderly people. BP was measured on left arm using digital automatic blood pressure monitor [OMRON]. Three measurements of BP were taken as per the WHO STEPS guidelines for physical measurements. Of the three measurements, the average of last two readings of Systolic Blood Pressure and Diastolic Blood Pressure was calculated and considered for assessment. The United States Seventh Joint National Committee on Detection, Evaluation and Treatment of Hypertension (JNC VII) blood pressure classification criteria was used. Individuals with systolic blood pressure ≥ 140 mmHg and/or diastolic blood pressure ≥ 90 mmHg or known hypertensive were classified as hypertensive.

All elderly people who were interviewed were weighed without foot wear, using an electronic weighing scale with an error range of ±100 grams. The weighing scale was regularly checked with known standard weights. A portable Stadiometer was used for measuring height, nearest to 1 cm, using standard procedure. Body Mass Index (BMI) was calculated as weight in kilograms divided by square of height in meters. Overweight and obesity was defined as BMI of ≥ 23 and ≥ 25 respectively (Normal BMI: 18.5–22.9 kg/m^2^ Overweight: 23.0–24.9 kg/m^2^, Obesity: >25 kg/m^2^) [23].

The research plan was submitted to Intuitional Ethics Committee at School of Public Health, SRM University and was approved. Informed written or verbal consent was obtained from all participants. In case of participants who were not literate, verbal consent was obtained after reading out the consent form to them and their verbal agreement was recorded by the interviewer in front of a witness. Data for all indicators were categorized and was analyzed using the IBM SPSS software for Windows, version 23.0 [24].

### Statistical analysis

Data were analyzed descriptively first. Age was categorized in three groups and recoded for further analysis. Descriptive statistics were calculated and presented as percentages for sociodemographic and health status assessment. The disability as domain specific functional limitation or difficulty in activity responses across age categories and gender were calculated presented as percentages. Descriptive age category wise distribution of responses to the items in six domains of WHO DAS2.0 questionnaire was calculated with cross tabs and presented in percentages. The Logistic regression analyses were conducted to estimate the association of age (category) progression with each item on the six domains of WHO Disability Assessment Schedule 2.0. Logistic regression was considered the appropriate statistical model because the dependent variables (difficulty experienced “yes” or “no”) were categorical and dichotomous. The estimated associations of age category with individual items on the six domains of WHODAS 2.0 are presented as odds ratios. The youngest age category of 60–64 years considered as reference and compared with older age category of 65–69 years and the oldest age category of 70 years and above. Significance level of 95% was considered for all estimations.

## Results

Table 1 describes the distribution of demographic characteristics; behavioral and physical measurements; health and social benefit indicators of the 506 elderly people in percentages. Women constituted a majority (58.89%) of the surveyed group in line with the assumption that women have longer life expectancy. Age was categorized to 5 year intervals; and in women the 60–64 age category was the majority (44.3%) with subsequent age categories gradually decreasing in number. This may reflect the natural phenomenon of age related increase in mortality. However, in men, the 70 and above age category was the majority (37.02%) with fewer people (27.88%) in the 60–64 age categories. The reason for increased mortality among relatively younger age category was not yet understood as part of this research. Majority of the women were widowed (67.45%) and living with children (49.33%). Among men majority were married (73.08%) and were living in a joint family set up (47.12%). A significant proportion of elderly women (15.44%) were living alone. Almost all the women (99.33%) had no formal schooling. A small proportion of men (11.06%) studied up to primary school. Majority of men (81.25%) and women (57.72%) were using tobacco. Majority of the men (80.77%) and nearly half of all women (47.32%) consumed alcohol. There were very few overweight or obese men or women in the surveyed group. Although, majority of men (58.17%) and nearly half of women (48.32%) were in the healthy Body Mass Index (BMI) category, a significant proportion of men (32.21%) and women (39.60%) were in underweight category. Almost half of all men (49.52%) and women (49.33%) reported moderate self-rated health scores. An equal and significant proportion of men (32.69%) and women (35.23%) reported bad self-rated health scores. Measurement of blood pressure revealed hypertension in nearly half of all men (53.85%) and an even higher proportion in women (63.42%). The reach of social benefits from government in the form of visits by health care worker was less than half in men (42.79%) and women (45.97%). With regard to old age pension from the state government, a similar scenario was seen with in men (48.56%). A slightly higher proportion of women (66.78%) received government pension.

**Table 1:** Distribution of demographics, behavioural and physical measurements and health and social benefit indicators

		Male	Female
		*N* = 208	N = 298
		%	%
Demographic information	Total surveyed	41.11	58.89
Age	60 ‐ 64	27.88	44.30
	65 ‐ 69	35.10	33.89
	70 and above	37.02	21.81
Marital status	Married (and) Cohabiting	73.08	29.53
	Divorced (or) Separated	1.92	3.02
	Widowed	25.00	67.45
Educational status	No Formal Schooling	87.02	99.33
	Up to Primary School	11.06	0.34
	More than Primary School	1.92	0.34
Living arrangement	Alone	2.88	15.44
	Spouse	25.00	12.42
	Children	22.12	49.33
	Joint family	47.12	17.11
	Relatives	2.88	5.70
Behavioural measurements			
	Currently using any tobacco	81.25	57.72
	Consumed Alcohol in last 30 days	80.77	47.32
Physical measurements			
Body mass index	Under weight	32.21	39.60
	Healthy	58.17	48.32
	Over weight	5.29	4.03
	Obese	3.37	1.68
Health indicators			
Self rated health	Good	17.79	15.44
	Moderate	49.52	49.33
	Bad	32.69	35.23
	Hypertension	53.85	63.42
Social benefit indicators			
	Visit by health worker	42.79	45.97
	Receiving old age pension	48.56	66.78

### Distribution of disability domains across age categories and gender

The distribution of disability as domain specific functional limitation or difficulty in activity responses across age categories and gender are presented in Table 2 as percentages. In the age category of 60–64, maximum functional limitation in elderly men and women were seen in performing house hold activities, followed by mobility. Activities of self-care and participation activities were the domains that measured the least functional limitation in both men and women. In comparison with men, women expressed more functional limitation for a majority of domains. As age advanced, functional limitation increased and were the highest for both house hold activity (from 81–93% to 98–100% for men and from (82–99% to 92–100% for women) and mobility (from 34–86% to 71–100% for men and from 46–90% to 86–96% for women) domains, for both men and women of age category 70 and above.

**Table 2:** Distribution of domain specific activity responses across Age categories and Gender

	Male			Female		
Age categories	60–64	65–69	70 & 70+	60–64	65–69	70 & 70+
N	58	73	77	132	101	65
Disability domains	%	%	%	%	%	%
Domain 1 Cognition						
Concentrating on doing something for 10 min?	50.00	42.47	67.53	53.79	57.43	69.23
Remembering to do important things?	50.00	53.42	63.64	62.12	62.38	61.54
Analysing and finding solutions to problems in day-to-day life?	24.14	35.62	58.44	39.39	55.45	69.23
Learning a new task, for example, learning how to get tp a new place?	65.52	68.49	90.91	78.03	93.07	87.69
Generally understanding what people say?	43.10	54.79	63.64	44.70	64.36	61.54
Starting and maintaining a conversation?	39.66	42.47	57.14	50.76	51.49	64.62
Domain 2 Mobility						
Standing for long periods such as 30 min?	79.31	78.08	97.40	90.15	92.08	96.92
Standing up from sitting down?	68.97	73.97	94.81	78.79	86.14	92.31
Moving around inside your home?	34.48	46.58	71.43	46.21	50.50	86.15
Getting out of your home?	63.79	58.90	88.31	64.39	73.27	90.77
Walking a long distance such as a kilometer [or equivalent]	86.21	84.93	100.00	90.91	94.06	95.38
Domain 3 Self Care						
Washing your whole body?	32.76	30.14	55.84	32.58	33.66	72.31
Getting dressed?	31.03	30.14	51.95	29.55	33.66	64.62
Eating?	27.59	17.81	49.35	17.42	25.74	58.46
Staying by yourself for a few days?	29.31	27.40	19.48	27.27	19.80	21.54
Domain 4 Getting Along With People						
Dealing with people you do not know?	51.72	50.68	51.95	59.85	62.38	58.46
Maintaining a freindship?	48.28	54.79	71.43	66.67	74.26	73.85
Getting along with people who are close to you?	65.52	64.38	70.13	72.73	67.33	64.62
Sexual activities?	74.14	73.97	66.23	43.18	22.77	9.23
Domain 5 Life Activities						
*Household activities*						
Taking care of your household responsibilities?	81.03	82.19	98.70	82.58	84.16	92.31
Doing your most important household tasks well?	84.48	89.04	100.00	94.70	85.15	95.38
Getting all the household work done that you needed to do ?	93.10	93.15	98.70	96.97	90.10	95.38
Getting your household work done as quickly as needed?	93.10	93.15	100.00	99.24	97.03	100.00
Domain 6 Participation						
How much of a problem did you have joining in community activities in the same way as anyone else can?	68.97	68.49	96.1	78.03	78.22	93.85
How much of a problem did you have because of barriers or hindrances in the world	65.52	69.86	92.21	72.73	78.22	90.77
around you?						
How much of a problem did you have living with dignity because of the attitudes and actions of others?	29.31	34.25	57.14	43.94	58.42	69.23
How much much time did you spend on your health condition or its consequences?	74.14	72.60	87.01	71.21	77.23	93.85
How much have you been emotionally affected by your health condition?	75.86	68.49	87.01	82.58	88.12	84.62
How much has your health been a drain on the financial resources of you or your family?	24.14	15.07	27.27	17.42	17.82	49.23
How much of a problem did your family have because of your health problems?	65.52	54.79	79.22	60.61	72.28	81.54
How much of a problem did you have in doing things by yourself for relaxation or pleasure?	68.97	71.23	94.81	81.06	89.11	95.38

### Association of disability with progressive age and gender

Table 3 describes the association of progressively increasing age (as age categories) with domain specific functional limitation across gender and between genders as odds ratios. For men, in comparison with age category of 60–64, activities that measured statistically highly significant increase in limitation for age category 70 and above were: (1) Learning a new task (5.6 times); (2) Generally understanding what people say (2.3 times); (3) Standing for long periods such as 30 min (9.7 times); (4) Standing up from sitting down (8.2 times); (5) Moving around inside home (4.7 times); (6) Getting out of home (4.2 times); (7) Joining in community activities (11.1 times); (8) Problem of participation because of barriers or hindrances in the world (3.2 times) and (9) Problem in doing things by oneself for relaxation (8.2 times). For women, in comparison with age category of 60–64, activities that measured statistically highly significant increase in limitation for age category 70 and above were: (1) Moving around inside your home (7.2 times); (2) Getting out of home (5 times); (3) Washing whole body (5.4 times); (4) Getting dressed (4.3 times) and (5) Eating (6.6 times). All mentioned values are highly significant. A 5 year increase in age did not result in any significant increase in functional limitation for both men and women. Although women had increased functional limitation in comparison to men as shown in Table 2, this increase was not found to be statistically significant except in the case of, learning a new task where the increase was highly significant (6.1 times).

**Table 3:** Association of Age Category with Disability Domains across Gender (with 60–64 Years as Reference) and between Gender

	Across Men			Across Women			Between Women and Men		
Disability Domains	Ref	65‐69 Yrs	70 & 70+ Yrs	Ref	65‐69 Yrs	70 & 70+ Yrs	60‐64 Yrs	65‐69 Yrs	70 & 70+ Yrs
Domain 1 Cognition									
Concentrating on doing something for 10 min?	1	0.48*(0.24‐0.97)	0.36**(0.18‐0.69)	1	1.16(0.69‐1.95)	1.93*(1.03‐3.62)	1.16(0.62‐2.15)	1.82(0.99‐3.36)	1.08(0.53‐2.20)
Remembering to do important things?	1	0.57(0.29‐1.14)	0.66(0.31‐1.26)	1	1.01(0.59‐1.73)	0.98(0.53‐1.80)	1.64(0.87‐3.05)	1.44(0.78‐2.66)	0.91(0.46‐1.80)
Analysing and finding solutions to problems in day‐to‐day life?	1	0.23**(0.11‐0.48)	0.39**(0.20‐0.76)	1	1.92(1.13‐3.24)	3.4*(1.84‐6.51)	2.04*(1.01‐4.09)	2.25*(1.21‐4.17)	1.6(0.79‐3.20)
Learning a new task, for example, learning how to get tp a new place?	1	1.14(0.55‐2.38)	5.26**(2.04‐13.57)	1	3.78(1.58‐9.04)	2.01(0.86‐4.68)	1.86(0.94‐3.69)	6.18**(2.47‐9.39)	0.71(0.24‐2.08)
Generally understanding what people say?	1	1.60(0.80‐3.21)	2.31**(1.15‐4.64)	1	2.23(1.31‐3.81)	1.98*(1.08‐3.63)	1.06(0.57‐1.98)	1.49(0.80‐2.75)	0.91(0.46‐1.80)
Starting and maintaining a conversation?	1	1.12(0.56‐2.27)	2.03*(1.01‐4.06)	1	1.03(0.61‐1.73)	1.77(0.96‐3.27)	1.56(0.83‐2.93)	1.43(0.78‐2.63)	1.37(0.69‐2.70)
Domain 2 Mobility									
Standing for long periods such as 30 min?	1	0.99(0.42‐2.33)	9.78**(2.09‐45.69)	1	1.27(0.51‐3.19)	3.44(0.75‐15.73)	2.38(1.01‐5.61)	3.06*(1.22‐7.67)	0.84(0.11‐6.13)
Standing up from sitting down?	1	1.35(0.63‐2.92)	8.21**(2.60‐25.94)	1	1.07(0.83‐3.38)	3.23*(1.19‐8.81)	1.67(0.83‐3.35)	2.07(0.95‐4.50)	0.65(0.16‐2.55)
Moving around inside your home?	1	1.66(0.81‐3.37)	4.75**(2.28‐9.89)	1	1.21(0.72‐2.04)	7.24**(3.31‐15.84)	1.63(0.86‐3.09)	1.19(0.65‐2.18)	2.49*(1.05‐5.88)
Getting out of your home?	1	0.81(0.40‐1.66)	4.29**(1.78‐10.31)	1	1.47(0.83‐2.61)	5.09**(2.04‐12.65)	1.09(0.57‐2.09)	1.99*(1.04‐3.78)	1.30(0.43‐3.87)
Walking a long distance such as a kilometer [or equivalent]	1	0.90(0.34‐2.41)		1	1.90(0.65‐5.58)	13.10(0.67‐14.29)	1.6(0.61‐4.15)	3.37*(1.11‐7.17)	
Domain 3 Self Care									
Washing your whole body?	1	0.89(0.42‐1.86)	2.60*(1.28‐5.28)	1	1.07(0.62‐1.85)	5.40**(2.81‐10.39)	0.99(0.51‐1.91)	1.19(0.62‐2.28)	2.07*(1.02‐4.18)
Getting dressed?	1	0.96(0.45‐2.03)	2.40*(1.18‐4.90)	1	1.23(0.70‐2.15)	4.36**(2.32‐8.19)	0.93(0.47‐1.82)	1.19(0.62‐2.29)	1.68(0.85‐3.32)
Eating?	1	0.57(0.25‐1.31)	2.56*(1.23‐5.30)	1	1.67(0.88‐3.14)	6.67**(3.42‐13.00)	0.54(0.26‐1.15)	1.62(0.76‐3.42)	1.44(0.79‐2.81)
Staying by yourself for a few days?	1	0.80(0.36‐1.78)	0.56(0.25‐1.29)	1	0.76(0.40‐1.45)	1.02(0.48‐2.14)	0.78(0.38‐1.6)	0.74(0.35‐1.55)	1.41(0.6‐3.33)
Domain 4 Getting Along With People									
Dealing with people you do not know?	1	0.96(0.48‐1.91)	1.01(0.51‐2.00)	1	1.11(0.65‐1.89)	0.94(0.52‐1.73)	1.39(0.74‐2.59)	1.61(0.87‐2.97)	1.30(0.66‐2.53)
Maintaining a freindship?	1	1.30(0.65‐2.59)	2.68*(1.31‐5.47)	1	1.44(0.81‐2.56)	1.41(0.73‐2.73)	2.14*(1.14‐4.02)	2.38*(1.25‐4.52)	1.12(0.53‐2.37)
Getting along with people who are close to you?	1	0.95(0.46‐1.96)	1.24(0.60‐2.56)	1	0.77(0.44‐1.36)	0.69(0.36‐1.29)	1.40(0.72‐2.72)	1.14(0.60‐2.15)	0.78(0.38‐1.57)
Sexual activities?	1			1	1.21(0.12‐12.25)			1.70(0.18‐16.08)	
Domain 5 Life Activities									
*Household activities*									
Taking care of your household responsibilities?	1	1.08(0.44‐2.63)	17.79*(2.22‐42.26)	1	1.12(0.56‐2.25)	2.53(0.92‐7.00)	1.10(0.5‐2.45)	1.15(0.51‐2.56)	0.16(0.01‐1.38)
Doing your most important household tasks well?	1	1.49(0.54‐4.15)		1	0.32*(0.13‐0.82)	1.16(0.29‐4.63)	3.28*(1.15‐9.29)	0.71(0.28‐1.76)	
Getting all the household work done that you needed to do ?	1	1.01(0.26‐3.94)		1	0.28*(0.09‐0.94)	0.65(0.14‐2.98)	2.37(0.57‐9.82)	0.67(0.21‐2.04)	
Getting your household work done as quickly as needed?	1	1.01(0.26‐3.94)		1	0.25(0.03‐2.43)		9.70*(1.06‐18.82)	2.40(0.55‐10.38)	
Domain 6 Participation									
How much of a problem did you have joining in community activities in the same way as anyone else can?	1	0.98(0.47‐2.06)	11.10**(3.08‐19.98)	1	1.01(0.54‐1.89)	4.29*(1.44‐12.80)	1.59(0.8‐3.19)	1.65(0.83‐3.27)	0.62(0.13‐2.86)
How much of a problem did you have because of barriers or hindrances in the world around you?	1	1.22(0.58‐2.55)	6.23**(2.31‐9.82)	1	1.35(0.73‐2.47)	3.62*(1.47‐9.28)	1.40(0.72‐2.72)	1.55(0.77‐3.08)	0.83(0.25‐2.71)
How much of a problem did you have living with dignity because of the attitudes and actions of others?	1	1.26(0.60‐2.64)	3.22*(1.56‐6.63)	1	1.77*(1.05‐2.99)	2.83*(1.51‐5.32)	1.91(0.98‐3.71)	2.70*(1.44‐5.03)	1.69(0.84‐3.37)
How much much time did you spend on your health condition or its consequences?	1	0.92(0.42‐2.02)	2.34(0.96‐5.68)	1	1.34(0.74‐2.43)	6.00*(2.04‐17.64)	0.88(0.44‐1.78)	1.28(0.64‐2.56)	2.28(0.67‐7.63)
How much have you been emotionally affected by your health condition?	1	0.69(0.32‐1.51)	2.13(0.87‐5.22)	1	1.50(0.70‐3.19)	1.11(0.49‐2.51)	1.57(0.74‐3.35)	3.41(1.56‐7.43)	0.82(0.31‐2.11)
How much has your health been a drain on the financial resources of you or your family?	1	0.56(0.21‐1.34)	1.20(0.55‐2.63)	1	1.03(0.52‐2.04)	1.06(0.49‐2.30)	0.66(0.31‐1.41)	1.24*(0.54‐2.80)	0.59(0.26‐1.32)
How much of a problem did your family have because of your health problems?	1	0.64(0.31‐1.30)	2.01(0.93‐4.34)	1	1.66(0.95‐2.91)	3.07*(1.47‐6.43)	0.83(0.43‐1.57)	2.15*(1.14‐4.05)	1.26(0.53‐2.96)
How much of a problem did you have in doing things by yourself for relaxation or pleasure?	1	1.11(0.53‐2.37)	8.21**(2.60‐15.94)	1	1.84(0.85‐3.95)	4.64*(1.34‐6.02)	2.00(0.98‐4.08)	3.30*(1.47‐7.39)	1.13(0.24‐5.25)

*P < 0.05; **P< 0.01

## Discussion

Disability is not a binary condition, but a multi-faceted, multi domain functional limitation. From this study it is evident that disability related functional limitations like mobility and performing house hold activities are more impaired in elderly tribal population than other limitations like cognition, self-care, getting along with people and social participation. Women comparatively reported higher disability across most domains of disability. Also it was evident that disability related functional limitations are highly progressive, however gender variation was not statistically significant. Similar studies reported similar results of higher functional disability in women [25]; higher effect of disability on mobility domain [26]; and a higher prevalence of hypertension [27]. Similarly, results also highlight the fact that even elderly people in tribals suffer from similar issues contrary to certain assumptions that functional disabilities are uncommon, because of their unique socio cultural life style [28]. A detailed primary evaluation of disability domains among significant numbers (i.e. 506 elderly people from 1349 households) of elderly population living in scattered tribal habitats, and hard to reach setting of Khammam District in Telangana are the strength of this study. Limitations include: (1) not assessing for simultaneous clinical disability for comparison; and (2) non- inclusion of elderly people who may be hospitalized or living in old age homes that could have led to under estimation.

Any discussion addressing the issue of disability related functional limitations of elderly population among tribals should be cognizant of the difficult environment that is predominantly open than built, and that of extreme resource and literacy constraints. For elderly with mobility limitations, difficult environment are associated with the majority of falls [29] and a fourfold increase in functional difficulty [30]. The special needs of women in these cultural settings should also be part of the solution. Present disability modifying mobility Assistive Devices (AD) can help elderly in overcoming functional limitations, attenuate functional decline, improve independence, help in community integration and enhance the quality of life [31]. However, these assistive devices are primarily designed for built indoor and outdoor environments and may not be helpful in open and naturally rugged environment [32]. Unsuitable for need and poorly designed assistive devices can lead to life threatening outcomes, abandonment of device, decreased independence and elderly being home bound [29, 30, 33]. Interplay between environment and disability modifying mobility Assistive Devices is an unexplored area for research and very scarce information is available at present. There is a clear and present need for disentangling and understanding the role of living environment, gender specific needs and cultural factors while designing and developing devices for outdoor mobility [32]. Literacy levels and resources for affordability are known to affect the knowledge, understanding and application of AD’s [30, 32]. Regular literacy based communication methods may not be helpful in reaching out to this study population with extremely low level of literacy. Traditional methods that involve arts or graphics or digital versions of traditional methods could be explored for sustained and effective communication on disability modifying means. Developing innovative assistive devices with locally available materials that are safe and effective should also be explored [34].

## Conclusion

This study highlights the fact that elderly population in tribal areas, much like the rest of the elderly population are affected by different domains of functional limitations and it is progressive. The needs, cultural sensitivities, and living environment of tribal population remain unique. Changing political landscape, the promise of better life, newer innovations, accessible and user friendly technologies should all converge around the needs of the poor and suffering elderly population among tribals. Along with the provision of regular monthly pension of INR 1500 to all elderly disabled people in tribal areas, the Government of Telangana should also support a research based complimentary process of understanding the local life, needs, local environment and means of communication beyond traditional methods to improve the life of elderly population living with disability in tribal areas.

## Abbreviations

AD: Assistive Devices
ADL: Activities of Daily Living
BMI: Body Mass Index
BP: Blood Pressure
INR: Indian Rupees
JNC VII: United States Seventh Joint National Committee on Detection, Evaluation and Treatment of Hypertension (JNC VII)
PPS: Probability Proportion to Size
TSP: Tribal Sub Plan area
WHO: World Health Organisation
WHO DAS 2.0: World Health Organisation Disability Assessment Schedule 2.0
